# Temporal Discounting Is Associated with an Increased Risk of Mortality among Community-Based Older Persons without Dementia

**DOI:** 10.1371/journal.pone.0067376

**Published:** 2013-06-27

**Authors:** Patricia A. Boyle, Lei Yu, Keith J. Gamble, David A. Bennett

**Affiliations:** 1 Rush University Medical Center, Rush Alzheimer’s Disease Center, Chicago, Illinois, United States of America; 2 Department of Behavioral Sciences, Rush University Medical Center, Chicago, Illinois, United States of America; 3 Department of Neurological Sciences, Rush University Medical Center, Chicago, Illinois, United States of America; 4 Department of Finance, DePaul University, Chicago, Illinois, United States of America; University of Missouri-Kansas City, United States of America

## Abstract

**Background:**

Temporal discounting is an important determinant of many health and financial outcomes, but we are not aware of studies that have examined the association of temporal discounting with mortality.

**Methods:**

Participants were 406 older persons without dementia from the Rush Memory and Aging Project, a longitudinal cohort study of aging. Temporal discounting was measured using standard preference elicitation questions. Individual discount rates were estimated using a well-established hyperbolic function and used to predict the risk of mortality during up to 5 years of follow-up.

**Results:**

The mean estimate of discounting was 0.45 (SD = 0.33, range: 0.08–0.90), with higher scores indicating a greater propensity to prefer smaller immediate rewards over larger but delayed ones. During up to 5 years of follow-up (mean = 3.6 years), 62 (15% of 406) persons died. In a proportional hazards model adjusted for age, sex, and education, temporal discounting was associated with an increased risk of mortality (HR = 1.103, 95% CI 1.024, 1.190, p = 0.010). Thus, a person with the highest discount rate (score = 0.90) was about twice more likely to die over the study period compared to a person with the lowest discount rate (score = 0.08). Further, the association of discounting with mortality persisted after adjustment for the level of global cognitive function, the burden of vascular risk factors and diseases, and an indicator of psychological well being (i.e., purpose in life).

**Conclusion:**

Temporal discounting is associated with an increased risk of mortality in old age after accounting for global cognitive function and indicators of physical and mental health.

## Introduction

Virtually all decisions involve choices between immediate and delayed rewards. From simple decisions, such as whether to take the elevator or stairs, to more complex ones, such as whether to undergo a difficult surgery with an uncertain outcome, we are inundated on a daily basis with an array of choices that require inter-temporal tradeoffs. Temporal discounting, the tendency to prefer smaller, immediate rewards over larger, delayed ones, has been the focus of numerous economic, behavioral economic, and psychology studies and is an important determinant of real world health and financial outcomes[1]–[7]. Individuals who tend to discount future rewards at a greater rate are more likely to be inactive, smoke, engage in risky alcohol and drug use, underutilize healthcare services, and be overweight compared to persons who exhibit less discounting [5], [8], [9]. In addition, those who discount future rewards tend to spend more, save less, make poorer investment decisions, and experience worse financial outcomes [2]–[4]. Given that temporal discounting is associated with poor decision making in domains critical to health and well being (i.e., health and financial matters), it stands to reason that discounting may also be related to mortality. That is, persons who exhibit poor health behaviors are more vulnerable to injury and illness over the lifespan, and those who make poor financial decisions likely experience more stress and related mental health issues, which may contribute to other health issues and ultimately mortality risk. To date, however, we are not aware of any study that has examined the association of discounting with the risk of mortality.

In this study, we tested the hypothesis that temporal discounting is associated with an increased risk of mortality among community based older persons without dementia. Participants were 406 older, non-demented persons from the Rush Memory and Aging Project, a longitudinal study of aging [10]. Temporal discounting was measured using standard preference elicitation questions in which participants were asked to make a hypothetical choice between an immediate, smaller payment of $1000 versus a delayed, larger payment that ranged from $1100 to 1500. Individual discount rates were estimated using a well-established function[2], [11]–[13], and proportional hazards models adjusted for age, sex, education were used to examine the association of temporal discounting with risk of mortality. In subsequent analyses, to examine the role of potential confounders or mediators of the relation between temporal discounting and mortality, we added terms to control for the influence of the starting level of global cognitive function and indicators of health status.

## Methods

### Participants

Participants were from the Memory and Aging Project, an ongoing longitudinal study of chronic conditions of aging that began in 1997; enrollment is ongoing [10]. Participation involves risk factor assessment, detailed annual clinical evaluations including medical history, neurological and neuropsychological examinations, and organ donation at death. The study was approved by the Institutional Review Board of Rush University Medical Center, and written informed consent was obtained following a detailed presentation of the risks and benefits associated with participation. Notably, in 2008, assessment of temporal discounting was started as part of a substudy that was also approved by the Institutional Review Board of Rush.

At the time of these analyses, 1560 eligible participants had completed the baseline evaluation for the parent study; of those, 516 died and 53 refused further participation in the parent project. Of the remaining 991 potentially eligible persons, 422 completed the temporal discounting assessment. We excluded 16 of these persons due to dementia, leaving 406 eligible for these analyses.

### Assessment of Temporal Discounting

Temporal discounting was assessed via 3 binary questions, following a standard preference elicitation protocol[2], [3], [11]–[13]. Participants were asked to choose between an immediate, smaller versus a delayed, larger payment, *e.g.,* “Which do you prefer, that you get $1000 in cash right now or $1100 in a year?” The current payment was fixed at $1000 and the delay period was fixed at one year for all questions. Delayed payments ranged from $1100, $1200 and $1500, with payment amounts varying across questions (*i.e.*, they did not escalate in sequence). The Cronbach’s alpha for this measure was 0.77, indicating adequate reliability.

### Clinical Diagnoses and Cognitive Function Testing

Clinical diagnoses were performed using a uniform process, as previously described in detail [10], and persons with dementia at the time of temporal discounting assessment were excluded from these analyses. Cognitive function was assessed via a battery of 21 tests, including the MMSE, but MMSE scores were used only to describe the cohort. One additional test, Complex Ideational Material, is used for diagnostic classification purposes only. Scores on the remaining 19 tests were used to create a summary measure of global cognitive function, as previously described. To compute the composite measure of global cognitive function, raw scores on each of the individual tests were converted to z-scores using the baseline mean and standard deviation of the entire cohort, and the z-scores of all 19 tests were averaged [10].

### Other Covariates

Other variables used in the analyses included age (based on date of birth and date of cognitive testing), sex (females coded as 0 and males as 1), education (years of schooling completed). Summary scores indicating each individual’s vascular risk burden (*i.e.*, the sum of hypertension, diabetes mellitus, and smoking, resulting in a score from 0–3 for each individual) and vascular disease burden (*i.e.*, the sum of heart attack, congestive heart failure, claudication, and stroke, resulting in a score from 0–4 for each individual) were computed on the basis of self-report questions, clinical examination, and medication inspection, as previously described(10). Finally, an indicator of psychological well-being, purpose in life (i.e., the tendency to derive meaning from life’s experiences and possess a sense of intentionality and goal directedness) was assessed using a 10-item scale derived from Ryff’s scales of Psychological Well-Being, as previously described [28]. For each of the 10 items, participants rated their level of agreement using a five point scale; scores on this measure are averaged to yield a total score, with higher scores indicating greater purpose in life.

### Determination of Vital Status

The autopsy rate of the Rush Memory and Aging Project exceeds 80%. Thus, for most participants, the exact date of death is known. Study participants also are contacted quarterly to determine vital status and changes in health, and death is occasionally learned of during quarterly contacts. Finally, research assistants regularly search the Social Security Death Index via the internet for the small number of persons lost to follow-up. At the time of these analyses, mortality data were accurate within three months.

## Data Analysis

### Data Analysis

Individual discount rates, defined here as 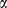
_,_ were estimated using the following well-established hyperbolic function[2], [12]–[14]:
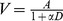
(1)where 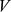 represents the discounted value of the delayed (later) reward 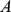 at delay 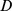
_._ The function shows that larger values of 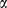 correspond to smaller values of 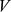.

Let observed outcome of a trial be denoted by 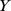, preference for the delayed reward by 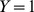 and the preference for the immediate reward by 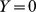. We hypothesized that the probability 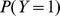 depends on the difference between the discounted delayed reward 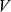 and the immediate reward 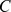. The odds of choosing the delayed reward over the immediate reward was formulated as
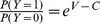
(2)


If 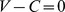
_,_ this indicates indifference between the immediate and delayed rewards. If 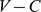 is positive, this indicates a preference for the delayed reward with odds greater than 1, and a negative 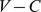 indicates a preference for the immediate reward. The discounting rate 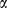 could be estimated from Eq (2).

Once individual discount rates were estimated, we examined the crude associations of temporal discounting with age, sex, and education, cognition and health conditions. Then, we examined the relation of temporal discounting with mortality using a proportional hazards model adjusted for age, sex, and education. Next, to examine the potential influence of cognition on the association of temporal discounting with mortality, we repeated the core model with an additional term for global cognitive function. Finally, we examined the contribution of health indices, particularly the burden of vascular risk factors and diseases. Model validation was performed graphically and analytically and there was no evidence of nonlinearity or non-proportionality. Programming was done in SAS [15].

## Results

### Descriptives

The mean discount rate was 0.45 (SD = 0.33, range: 0.08–0.90), with larger values indicating greater discounting. Temporal discounting was negatively correlated with education (r = −0.185, p<0.001), such that more educated persons exhibited less discounting, and women discounted more than men (p = 0.009); discounting was not significantly associated with age. Temporal discounting was negatively associated with global cognition (r = −0.142, p = 0.004).

### Temporal Discounting and Mortality

Over up to 5 years of follow-up (mean = 3.6), 62 (15% of 406) persons died. Table 1 provides crude data at baseline on those who died and those who survived. Those who died were older and exhibited greater temporal discounting compared to survivors. In addition, those who died had lower levels of global cognitive function and a greater burden of vascular health conditions.

**Table 1:** Baseline Characteristics of Participants Who Survived or Died.

Characteristic*	Survived (n = 344)	Died (n = 62)	P Value
Age	82.6 (6.63)	86.4 (5.54)	<0.001
Sex (% Female)	265 (77.0%)	42 (67.7%)	0.117
Race (% White)	327 (95.1%)	61 (98.4)	0.331
Education	15.3 (3.09)	14.5 (2.63)	0.067
Temporal discounting	0.43 (0.32)	0.56 (0.35)	0.012
Global cognition	0.28 (0.517)	−0.06 (0.535)	<0.001
Vascular risk factors	1.14 (0.780)	1.44 (0.822)	0.012
Vascular disease	0.53 (0.779)	0.95 (1.035)	0.001

* Mean values are presented unless otherwise noted and statistical significance is based on t-tests (or Mann-Whitney Wilcoxon rank sum) or Chi-Square tests, as appropriate.

**Figure 1:**
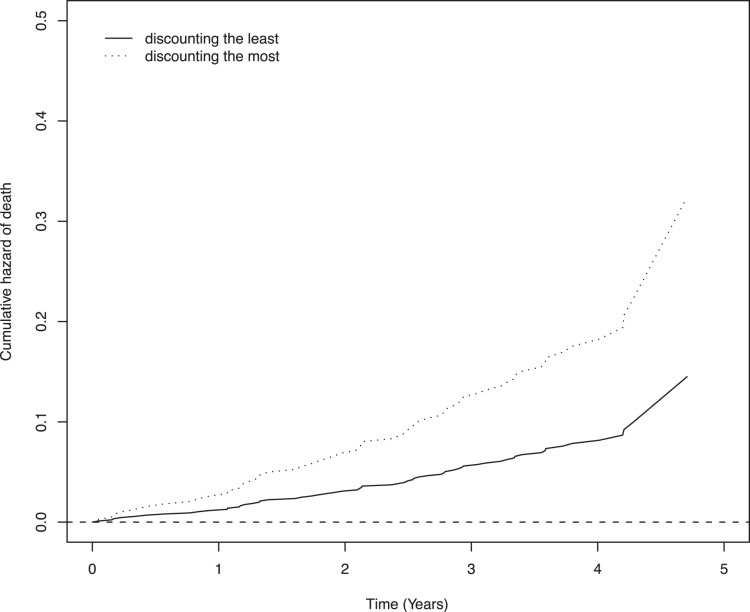
Cumulative hazard of mortality for participants with greater (dotted line) vs. lower levels of discounting (solid line) temporal discounting derived from a model adjusted for age, sex and education.

We examined the association of temporal discounting with the risk of mortality via a series of proportional hazards models adjusted for age, sex, and education. In the initial analysis, the risk of mortality increased about 10% for each 0.1 unit increase in temporal discounting (HR = 1.103, 95% CI 1.024, 1.190, p = 0.010). Thus, a person with the highest discount rate (score = 0.897) was about twice more likely to die compared to a person with the lowest discount rate (score = 0.079).

Next, because it is well known that cognitive function and health status are associated with the risk of mortality in old age and temporal discounting is related to both cognitive function and health, we examined whether the association of temporal discounting with mortality persisted after adjustment for global cognitive function and indices of health. Thus, we first repeated the initial proportional hazard model described above but included an additional term for the starting level of global cognitive function, which was measured using 19 cognitive tests. The association of temporal discounting with mortality persisted and was only slightly reduced in this analysis (HR 1.090, 95% CI 1.011, 1.174, p = 0.025), suggesting that it is relatively independent of global cognition. In addition, we repeated the core model with additional terms for vascular risk factors and diseases. Again, the association of discounting with mortality persisted and was essentially unchanged (HR 1.092, 95% CI 1.012, 1.177, p = 0.022). Finally, we repeated the core model with a term for an indicator of psychological well being (i.e., purpose in life); the association persisted (HR 1.083, 95% CI 1.003–1.168, p = 0.037).

### Secondary Analyses

To further examine the robustness of the findings reported above, we examined response patterns and conducted additional analyses to determine whether the above findings persisted when examining response patterns directly. That is, for this analysis, we grouped participants into those who always took the later payment, those always took the current payment, and those who took the current payment when the later payment was only incrementally bigger than the current payment (i.e., $1000 today versus $1100 in one year) and then switched to the later payment when that payment increased to $1200 or $1500 in one year. In doing so, we also identified 19 persons who exhibited inconsistent response patterns (e.g., they chose a later payment of $1100 but then took the current payment when the later payment was $1200 or $1500). After excluding inconsistent responders, we examined the relation of response patterns with mortality and all findings persisted even after adjustment for the relevant covariates (data not shown).

## Discussion

We examined the association of temporal discounting with mortality in more than 400 community-dwelling older persons without dementia. Temporal discounting was associated with a substantially increased risk of death; specifically, a person with the highest discount rate was about twice more likely to die over the study period compared to a person with the lowest discount rate. Further, the association of temporal discounting with mortality persisted after adjustment for the starting level of global cognitive function as well as the burden of vascular risk factors and vascular diseases. These findings suggest that temporal discounting warrants additional research focus as a potentially early and important harbinger of adverse health outcomes in old age.

Temporal discounting has long been recognized as an important determinant of health and financial behaviors, both of which are essential to maintaining independence and well-being across the lifespan[1]–[9], [16]. Although it stands to reason that temporal discounting would predict adverse health consequences, we are not aware of any longitudinal study that has directly examined this question. This reflects an important gap in knowledge. Further, temporal discounting may be critically important in aging, the time when many consequential decisions are made just as cognitive function and other abilities deteriorate and the burden of disease increases. Relatively little is known about discounting among older persons; some cross sectional studies have sought to clarify whether discounting is greater or lower among older persons compared to younger persons, but findings are mixed[17]–[21]. Whether or not discounting changes with age, the behaviors associated with discounting may be most damaging in old age; for example, poor health behaviors such as smoking and drinking may be particularly harmful in the later years, as physical function deteriorates. Notably, in this study, we examined the potential role of smoking (among other vascular risk factors) as well as vascular diseases, and the association of discounting with mortality persisted even after adjustment for those risk factors and conditions. This suggests that temporal discounting may reflect a broad construct that works via mechanisms other than physical health status to influence mortality.

Another important consideration in understanding the link between temporal discounting and mortality is the role of cognitive ability, as some prior studies have demonstrated associations between discounting and indicators of cognitive ability[22]–[24]. Cognitive ability also is widely recognized as a strong predictor of mortality. Here, we excluded persons with overt dementia from the analyses. Further, we controlled for starting level of cognition using a detailed battery of 19 cognitive tests and the association of temporal discounting with mortality persisted. Thus, the effect of temporal discounting on risk of death was above and beyond level of cognition. This finding supports the validity of temporal discounting as a construct that is relatively distinct from cognition, an idea that has been advanced in prior studies showing that discounting predicts important real world behaviors after controlling for cognitive ability [3], [22]. We conceptualize discounting as an indicator of impulsivity, at least to some degree, and we suspect that it can dramatically affect decision making and health outcomes because cognitive ability alone is insufficient to promote good decision making. For example, there are many highly intelligent adults that drink and drive, smoke, are physically inactive, or make other poor choices. All of these behaviors have a range of adverse consequences and reflect, in varying degrees, the tendency to exhibit an impulsive preference for immediate rewards over longer-term but potentially greater rewards. Additional research is needed to better understand discounting in old age and to determine whether temporal discounting is related to other important outcomes.

The basis of the association of temporal discounting with mortality is unknown. One possibility is that this association is the result of co-morbidities due to a history of poor decision making in domains that affect health. That is, older persons who exhibited greater discounting likely engaged in poorer health behaviors over the lifespan and may have been sicker and therefore at greater risk of death. However, as noted above, controlling for disease burden did not eliminate the finding. It also is possible that persons who exhibited greater discounting made poorer financial decisions and experienced more stress and related mental health problems; the British Household Panel Survey (BHPS) found that higher financial capability, which is related to discounting, was associated with better self-reported mental and physical health [27], and we have previously shown that a related construct, psychological well-being (i.e., purpose in life), is associated with the risk of mortality [28]. However, the association of discounting with mortality persisted after controlling for purpose in life. Another possibility is that future time perspective, a construct that our measure of purpose in life assesses to a limited degree, underlies the association of discounting with mortality; that is, as individuals age and recognize that mortality is approaching simply as a function of age, they may discount delayed future rewards because of the sense of limited time left in life. Whereas this may be an age-appropriate phenomenon, we unfortunately did not have data to address future time perspective. Finally, another possibility is that common, age-related neuropathologies (i.e., Alzheimer disease pathology and cerebrovascular disease), which are frequently found in the brains of older persons with and without dementia and are associated with subtle cognitive decrements even among persons without dementia, also affect impulsivity and related behaviors that support decision making [25], [26]. Thus, the tendency to discount future rewards may increase as age-related pathology accumulates in the brain and compromises behavior regulation. Future studies are needed to elucidate the neurobiologic basis of the association of temporal discounting with mortality, but we suspect that the association is the result of disease processes that either result from or contribute to poor decision making in old age.

This study has several strengths, including the assessment of temporal discounting in a large group of community-dwelling older persons who underwent a uniform clinical evaluation and in whom widely accepted criteria were used to exclude persons with dementia. In addition, we examined potential confounders of the association of temporal discounting with mortality, including the level of global cognitive function measured via a well-established and detailed battery of tests and two summary measures of disease burden. Limitations include the selected nature of the cohort and the use of only three items to assess discounting. The use of only three questions limited the range of scores on the discounting measure as well as our ability to distinguish discount rates among those who always took the current payment and among those who always took the later payment; thus, the numerical values of the discounting measure were constrained and this may limit the generalizability of findings. Further, because we only varied the payments and did not vary the time delay, we were not able to analyze the data using area under the curve (AUC), a non-theoretical approach that is increasingly used in the discounting literature [29]. In addition, the duration of follow-up was relatively short, although the results were significant and robust. Finally, we did not examine how temporal discounting changes with age. Future studies are needed to better understand the trajectory of temporal discounting in aging and to further examine the association of temporal discounting with additional health outcomes.
